# Catestatin as a Target for Treatment of Inflammatory Diseases

**DOI:** 10.3389/fimmu.2018.02199

**Published:** 2018-10-04

**Authors:** Elke M. Muntjewerff, Gina Dunkel, Mara J. T. Nicolasen, Sushil K. Mahata, Geert van den Bogaart

**Affiliations:** ^1^Department of Tumor Immunology, Radboud Institute for Molecular Life Sciences, Radboud University Medical Center, Nijmegen, Netherlands; ^2^VA San Diego Healthcare System, San Diego, CA, United States; ^3^Department of Medicine, University of California at San Diego, La Jolla, CA, United States; ^4^Department of Molecular Immunology, Groningen Biomolecular Sciences and Biotechnology Institute, University of Groningen, Groningen, Netherlands

**Keywords:** catestatin, immune modulation, macrophages, anti-inflammatory, inflammatory disease, chromogranin A

## Abstract

It is increasingly clear that inflammatory diseases and cancers are influenced by cleavage products of the pro-hormone chromogranin A (CgA), such as the 21-amino acids long catestatin (CST). The goal of this review is to provide an overview of the anti-inflammatory effects of CST and its mechanism of action. We discuss evidence proving that CST and its precursor CgA are crucial for maintaining metabolic and immune homeostasis. CST could reduce inflammation in various mouse models for diabetes, colitis and atherosclerosis. In these mouse models, CST treatment resulted in less infiltration of immune cells in affected tissues, although *in vitro* monocyte migration was increased by CST. Both *in vivo* and *in vitro*, CST can shift macrophage differentiation from a pro- to an anti-inflammatory phenotype. Thus, the concept is emerging that CST plays a role in tissue homeostasis by regulating immune cell infiltration and macrophage differentiation. These findings warrant studying the effects of CST in humans and make it an interesting therapeutic target for treatment and/or diagnosis of various metabolic and immune diseases.

## Introduction

Inflammation-based diseases, such as chronic inflammation (Type 2 diabetes Mellitus (T2DM) and colitis), auto-immune diseases (rheumatoid arthritis (RA) and systemic lupus erythematosus (SLE)), hypertension, tumor metastasis and development of severe cancers (myeloma, neuroendocrine tumors, lung, and breast cancer) (1–3) are major health problems. For instance, 415 million adults were globally affected by T2DM in 2015 and this caused 5.0 million deaths (4). For RA the current mortality rate is 2.2 million (5) and for SLE comorbidities including infection and cardiac malfunction account for 29% of all deaths (4, 6). The second leading cause of death worldwide is cancer and 1 in 6 people die to cancer, accounting for 8.8 million deaths in 2015 (4). The prevalence of all these diseases is increasing and, in many cases, sufficient therapies are not available. Recently, an interest in utilizing the body's own molecules to treat these diseases arose. An interesting candidate is the pro-hormone chromogranin A (CgA), which contributes to a balanced immune response. CgA is proteolytically cleaved, both intracellularly as well as extracellularly after its release, and this gives rise to several peptides (7, 8). These peptides exert a broad spectrum of regulatory functions among the metabolic, endocrine, cardiovascular and immune systems (9). It is becoming increasingly clear that one of these cleavage products, the bio-active peptide catestatin (CST: hCgA_352−372_) (10, 11), is particularly of interest, since it suppresses tissue inflammation and affects the immune system. Indeed the concept is emerging that CST plays an immunomodulatory role in macrophage differentiation and monocyte migration. This review will focus on the relatively new concept of modulating innate immunity by targeting CST, which may find applications in treatment of various inflammatory based diseases and cancer (1–3). We will review the effect of CST on infiltrating immune cells, tissue homeostasis and the role of CST in disease. Moreover, we will discuss remaining outstanding questions about the effects and molecular targets of CST, as well as further directions in research and therapeutic applications.

## Cleavage products of the pro-hormone chromogranin A

### Chromogranin a (CgA)

The human CgA gene is located on chromosome 14 (12, 13) and codes for a 439 amino acids long protein (14). As member of the granin family, CgA is characterized by an acidic pI, heat stability and 8-10 pairs of dibasic cleavage sites (15). Moreover, this 49 kDa protein has the capacity to form aggregates and the ability to bind calcium (Ca^2+^) with a high capacity, but low affinity (16). CgA was first identified as an acidic protein co-stored and co-released with ATP and catecholamines in chromaffin granules of neuroendocrine cells in the adrenal medulla (17, 18). CgA facilitates the storage in these granules of catecholamines and ATP at hyperosmotic concentrations in a non-diffusible form (17–21). Thereby CgA contributes to the biogenesis of secretory granules packed with condensed proteins, mostly (pro) hormones (22, 23) via recruitment of proteins involved in the formation and trafficking of vesicles, such as cytoskeleton-, GTP-, and Ca^2+^-binding proteins (24). The secretory granules route toward the cell periphery, where they mature and undergo calcium-controlled exocytosis (25–27). Upon an increase in Ca^2+^ concentration, CgA is co-released simultaneously with the stored hormones of the secretory granules via exocytosis (25–27). CgA is not only present in chromaffin cells, but has been detected in other secretory vesicles of endocrine, neuroendocrine and neuronal tissues (28–31) as well as in keratinocytes (32), myocardial cells (33–35), endothelial cells (36, 37), and macrophages (36). Interestingly, CgA is also present in cells of the pancreatic islet, secretory granules of glucagon containing α-cells and insulin producing β-cells, and may thereby modulate glucose metabolism (31, 38–42). This makes CgA particularly interesting in the context of metabolic diseases, such as diabetes. Patients suffering from carcinoids or other neuroendocrine tumors (25, 43–47), heart failure, renal failure, hypertension, RA, and IBD (48–54) display increased levels of circulating CgA, implicating an important role of CgA to influence human health and disease (3).

### Cleavage products of CgA

**Figure 1:**
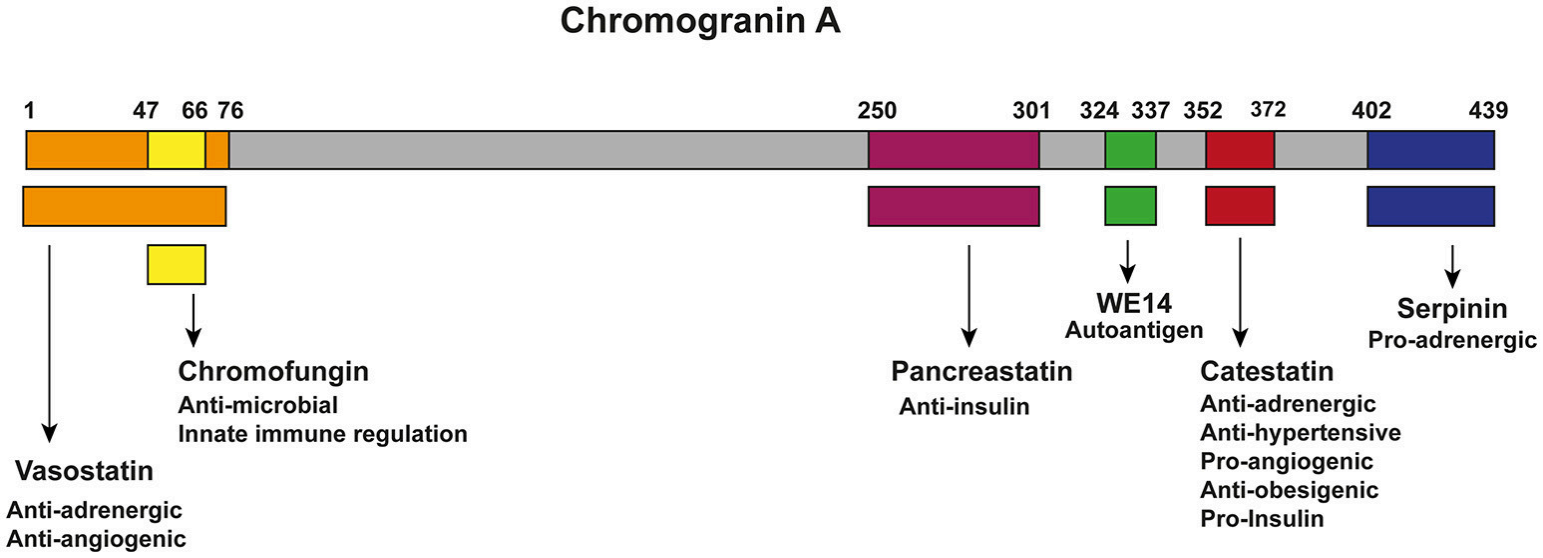
Chromogranin A and its bio-active peptides. Cleavage of CgA gives rise to six known biological active peptides: vasostatin (Orange; hCgA_1−76_), which has anti-adrenergic and anti-angiogenic functions (55–57); Chromofungin (Yellow; hCgA_47−66_) has antimicrobial effects as well as effects on innate immune regulation; pacreastatin (Purple; PST; hCgA_250−301_), which has anti-insulin functions (42, 58, 59); WE-14 (green; hCgA_324−337_), which acts as an autoantigen for the highly diabetogenic CD4^+^ T cell clones (60–62); CST (Red; hCgA_352−372_), which has pro-insulin, anti-obesigenic (63), pro-angiogenic (64, 65), anti-adrenergic, anti-hypersensitive (65–68), cardiomodulatory (8, 69–74) and anti-inflammatory functions (8, 32, 36, 75–78); serpinin (blue; hCgA_402−439_), which is pro-adrenergic and regulates granule biogenesis and acts as a myocardial ß agonist (79, 80).

CgA can be proteolytically processed in various tissues and thereby serves as a precursor for several biological active peptides (Figure 1). The cleavage of CgA at its dibasic sites is performed by intra-granular and extra-cellular proteases, such as prohormone convertases 1 (PC1) (81), PC2 (81), furin (81), cysteine protease cathepsin L (CTSL) (82), the serine proteases plasmin (83, 84) and thrombin (85), as well as by kallikrein (86). Depending on the cleavage sites, post-translational modifications (glycosylation and phosphorylation) and proteolytic processing, CgA can result in the following six biological active peptides (9, 87). The first peptide identified was pancreastatin (PST) (hCgA_250−301_), which has an opposing effect to insulin (42, 58, 59). WE-14 (hCgA_324−337_) was identified in midgut carcinoid tumors and acts as an antigen for the highly diabetogenic CD4^+^ T cell clones (60–62). Chromofungin (hCgA_47−66_) has antimicrobial effects as well as effects on innate immune regulation (88, 89). Vasostatin (hCgA_1−76_) has a vasodilative and anti-angiogenic as well as antiadrenergic functions (55–57). Serpinin (hCgA_402−439_) regulates granule biogenesis (79) and acts as a myocardial ß agonist (80). Finally, the pleotropic peptide CST (hCgA_352−372_) has mainly anti-inflammatory effects (8, 90) and is the central focus of this review. The N-terminal 15 amino acid domain of bovine CST is called Cateslytin (bCgA_344−358_), which is the active domain of CST (76, 91). CgA is unique as several of its peptides exhibit opposing counter-regulatory effects for fine-tuning and maintaining metabolic homeostasis. As for cardiac function, this is regulated in rodents by the pro-adrenergic peptide serpinin (80) and both antiadrenergic peptides vasostatin and CST (66, 92). Likewise, angiogenesis is controlled by the antiangiogenic peptide vasostatin (85, 93) and the proangiogenic peptide CST (64, 85). Similarly, glucose homeostasis is maintained by pancreastatin (42, 58, 59, 94), which is an anti-insulin peptide and CST, which is a pro-insulin peptide (75). Although CgA processing has been reported to occur intracellularly inside the hormone-storage vesicles and extracellularly after its release in the blood, no systematic studies have been conducted to determine whether several proteolytic enzymes act at the same time to liberate all of the CgA peptides or act at different sites at different times in a tissue-specific manner. In addition, no attempts have been made so far to assess whether CgA peptides are generated in equal molar amounts or generated in response to physiological demands in different tissues. However, it has been reported that circulating concentrations of CgA peptides are different. For example, plasma vasostatin levels vary from 0.3 to 0.4 nM and CST circulates at 0.03 to 1.5 nM concentrations (9), which might represent different degrees of processing or rates of clearance from the circulation.

### The pleotropic peptide catestatin (CST)

The CgA plasma levels range from 0.5 to 1 nM (9), whereas the physiological blood levels of CST range from 0.03 to 1.5 nM in healthy subjects (9, 95). At first CST was identified as a potent inhibitor of nicotine induced catecholamine release. As CST is secreted together with catecholamines, it can thereby function as an autocrine negative feedback-loop self-limiting further catecholamine secretion (10, 96, 97). Later, CST was found to play a role in the regulation of hypertension (65–68) and cardiac functions (8, 69–74), as well as in promoting angiogenesis (64, 85), decreasing obesity (63) and regulating innate immunity (8, 32, 36, 75–78). In line with this, alternated plasma levels of CST or its prohormone CgA have been observed in the context of various diseases. Plasma levels of CST are reduced in patients suffering from T2DM and hypertension (75, 95, 98), whereas elevated levels of the pro-hormone CgA have been detected in the plasma of patients with neuroendocrine tumors (25), hypertension (99, 100) and various inflammatory diseases, such as RA (6, 101, 102), SLE (6), inflammatory bowel disease (IBD) (53, 54, 103–105) as well as T1DM and T2DM (62, 106–109). This suggests that the lower levels of CST are caused by a dysregulation of proteolytic processing of CgA (98). The balance between processed peptides seems also important to counteract effects of the bio-active peptides. Since vasostatin has an anti-angiogenic effect (55–57), this might counteract the pro-angiogenic effect of CST (64, 85). Moreover, CgA-knockout (CgA-KO) mice develop an obese phenotype (42) as well as severe hypertension which could be rescued by intra-peritoneal injections of CST (67). Since hypertension is linked to diabetes, heart diseases and psoriasis (110), this indicates that CST might be important in various severe diseases. These findings support the hypothesis that the impaired processing of CgA might lead to lower CST levels which contributes to disease development. They also warrant further studies to elucidate the effects and mechanisms of CgA and its bio-active peptide products.

## CST contributes to maintenance of metabolic and immune homeostasis

### CST effects on metabolism

In addition to its anti-inflammatory effects, CST also affects metabolism. Opposite to insulin, CST inhibits lipogenesis and increases lipolysis in adipose tissue by inhibition of the α2-adrenergic receptor and by enhancing leptin signaling (63). Simultaneously, it stimulates fatty acid uptake and breakdown in the liver, as reflected by increased expression of the genes involved in fatty acid oxidation upon intra-peritoneal CST injections in mice (63). In line with this, CST injections in CgA-KO mice resulted in decreased triglyceride levels in the plasma and reduced fat depot sizes by ~25% (63). These findings indicate that CST promotes lipid flux from adipose tissue to the liver for beta oxidation, which might explain the frequently observed weight gain in patients with inflammatory diseases, as these patients have lower plasma levels of CST (75, 95, 98).

Besides the effect on lipid metabolism, intra-peritoneal administration of CST improved glucose and insulin tolerance in Diet-induced obese (DIO) mice and insulin-resistant systemic CST-KO mice, that express a truncated version of CgA (75). This could be due to CST inhibiting gluconeogenesis in the liver, thereby lowering the production and release of glucose in the blood (75). This effect of CST could be mediated by the modulation of Kupffer-cells and monocyte-derived macrophages, since the effects of their cytokines are linked to glucose and insulin metabolism (75, 111) and for instance neutralization of TNF-α improves insulin sensitivity (112). Thus, CST can promote lipid and glucose metabolism, and thereby might help to prevent obesity and maintain homeostasis of metabolic functions (7, 63, 75). Although CST immunoreactivity has been detected in carcinoid tumors of the appendix, bronchus, stomach, small bowel and large bowel (113), its effects on cancer metabolism is yet to be investigated. However, insulin has been reported to promote cancer metabolism by upregulating pyruvate kinase M2 isoform (PKM2) expression and decreasing its activity, eventuating in amplification of cancer-metabolism-specific parameters like glucose uptake, lactate production, glycolytic pooling and macromolecular synthesis (114). In addition, several reports reveal increased cancer risk under hyperinsulinemic condition (115, 116). Since CST decreases insulin level in hyperinsulinemic as well as insulin-resistant DIO and CST-KO mice (75), we expect that CST would decrease tumor growth by decreasing expression of PKM2 and increasing its activity, which requires experimental validation. Interestingly, PST, another cleavage product of CgA, counteracts the metabolic and insulin sensitizing effects of CST (75). These anti-insulin actions of PST are likely important in maintaining homeostasis in glucose metabolism (7, 42, 94). The exact regulation of the proteolytic generation of CST and PST remains to be elucidated, but it could be coupled to metabolism via glycosylation because hyper-glycosylation of CgA is known to lead to reduced levels of CST (117). Thereby, the generation of CgA cleavage products might be regulated by sugar levels and this might play a role in progression of metabolic diseases, as for instance the increased blood glucose levels in T2DM might promote glycosylation of CgA.

### CST regulates immune homeostasis

**Figure 2:**
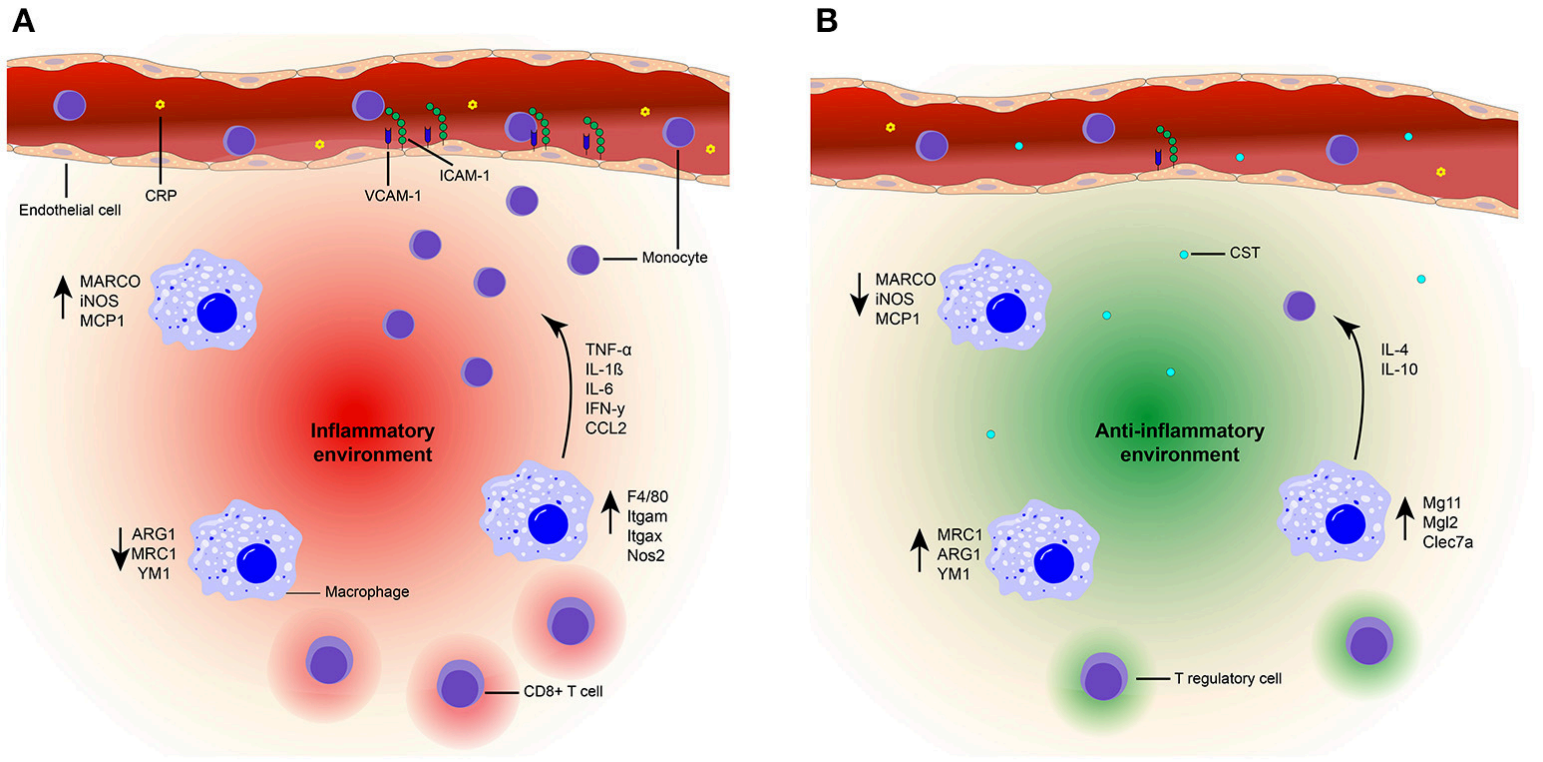
Model of the suppressive effects of CST on inflammation. **(A)** Inflammatory state. High plasma levels of CRP (yellow) are present in an inflammatory state. Due to the presence of chemokines (CCL2) and upregulation of integrin ligands [ICAM-1 (green) and VCAM-1 (blue)] on the endothelial cells (orange), increased monocyte (purple) infiltration is present at the inflammation site. An inflammatory environment (red) is created by the upregulation of pro-inflammatory markers (F4/80, Itgam, Itgax, NOS2, MARCO, iNOS, MCP1) in macrophages (blue). The production of inflammatory cytokines is also increased (TNF-a, IL-1β, IL-6, IFN-γ) and infiltration of CD8^+^ T cells occurs. **(B)** CST treated state. Treatment with CST (light blue) results in lower levels of circulating CRP (78, 120) and reduced expression of integrin ligands (36). Monocyte infiltration is reduced (36, 75, 78, 121) and macrophages are polarized toward an anti-inflammatory phenotype, characterized by upregulation of anti-inflammatory markers [MRC1, ARG1, YM1, Mg11, Mgl2, Clec7a (36, 75, 121)] and increased production of IL-10 and IL-4 (75, 121). Moreover, expression of pro-inflammatory genes and cytokines are reduced (36, 75, 78, 121). There will be no infiltration of CD8^+^ T cells and Tregs might be present. Together, this contributes to an anti-inflammatory environment (green) and improved tissue architecture (36, 78, 121).

CST contributes to the defense against infections in several ways (76, 77, 118). An initial study utilized 15 amino acids from the N-terminal end of bovine CST or cateslytin to demonstrate their antimicrobial activities (76). First, CST can directly act on invading microbes, as CST can penetrate the membrane of bacteria and fungi. At relatively high concentrations (>μM) it thereby directly can impair the growth of fungal pathogens (76). Moreover, CST can induce lysis of bacteria and helps to protect against infections following skin injuries in mice (32). Second, at least *in vitro*, CST can result in activation of neutrophils and mast cells which contribute to innate immune responses to infections (76–78, 118, 119). These effects may be restricted to local high CST concentrations, whereas systemic anti-inflammatory effects of CST have been best described in autoimmune diseases (Figure 2).

In a colitis mouse model, intra-rectal injections with CST resulted in decreased serum levels of the acute phase reactant C-reactive protein (CRP) (78, 120) and suppressed activity of myeloperoxidase (MPO), which is a marker for granulocyte infiltration (78). As a result of these injections, the tissue architecture of the colon improved (78, 121). Moreover, in atherosclerotic mice (apolipoprotein E-deficient mice), intraperitoneal injection followed by continuous subcutaneous CST infusion significantly retarded atherosclerotic lesions by 40% in the entire surface area of the aorta (36). In both colitis and atherosclerosis models, the prevalence of macrophages and monocytes in inflamed tissues was reduced following administration of CST (36, 78, 121), thereby supporting the anti-inflammatory effects of CST. In DIO mice, the intra-peritoneal injection of CST inhibited the infiltration of monocytes in the liver and reduced CC-chemokine ligand 2 (CCL2)-induced chemotaxis of peritoneal macrophages (75). The molecular mechanisms by which CST affects monocyte and macrophage migration are still unclear. One possibility is that CST directly affects leukocyte migration. This is shown for monocytes, although in this case already low concentrations of CST (nM) promoted migration in an *in vitro* chemotaxis assay (122). The reasons underlying this discrepancy between *in vitro* and *in vivo* experiments is unclear, but could be due to CST affecting other chemokines (such as CCL2) present in the *in vivo* situation. Moreover, CST is also pro-angiogenic (64, 85) which might reduce its anti-inflammatory effect when present on its own. Another possibility is that CST affects the integrins that affect leukocyte extravasation. This possibility is supported by the finding that CST can reduce expression levels of the integrin ligands intracellular adhesion molecule 1 (ICAM-1) and vascular CAM-1 (VCAM-1) in endothelial cells, which correlate with lymphocyte extravasation (36). Finally, CST might reduce monocyte infiltration in inflammatory tissues by lowering the production of pro-inflammatory cytokines and chemokines by macrophages due to altered macrophage differentiation (75, 78).

Considering the effects of CST treatment on THP-1 cells (a human monocyte cell line), it seems that CST does not affect the overall differentiation of monocytes to macrophages. This was shown by consistent expression of the macrophage marker CD68 by THP-1 cells under CST treatment. However, CST steers the polarization of differentiation into less pro- and more anti-inflammatory phenotypes (36). CST treatment of THP-1 derived macrophages resulted in elevated levels of anti-inflammatory macrophage markers (mannose receptor C-type 1, (MRC1)) and reduced levels of pro-inflammatory macrophage markers (macrophage receptor with collagenous domain, (MARCO)) (36). Additionally, the gene expression levels of the pro-inflammatory macrophage markers inducible nitric oxygen synthase (*iNOS*) and monocyte chemoattractant protein 1 (*Mcp1*) were reduced upon intra-rectal injection of CST in a reactivated colitis mouse model, as well as *in vitro* in LPS stimulated peritoneal and colon macrophages (78, 121). In both the mouse model and *in vitro*, CST treatment resulted in decreased levels of pro-inflammatory cytokines (IL-6, IL-1β, TNF-α) (78, 121). In the reactivated colitis mice model, CST promoted expression of several anti-inflammatory genes (*IL-10, Arg1*, and *Ym1*) of the macrophages in the colon (121). Moreover, a reduction of pro-inflammatory gene expression (*TNF-*α*, F4/80, Itgam, Itgax, Ifng, Nos2*, and *Ccl2*) was detected in isolated Kupffer cells and monocyte derived macrophages of DIO mice after intra-peritoneal injections with CST, whereas levels of anti-inflammatory genes were increased (*IL-10, Mgl1, IL-4, Arg1*, and *Mrc1*) (75). These results could be confirmed in isolated macrophages treated *in vitro* with CST (75). In the DIO mouse model, intra-peritoneal injections with CST also reduced plasma levels of pro-inflammatory cytokines and chemokines (TNF-α, INF-γ, and CCL2) (75). Taken together, these findings indicate that CST shifts macrophage differentiation from a pro- to a more anti-inflammatory phenotype. Since adipose tissue macrophages (ATMs) have antigen-presenting capacities this shift could influence the adaptive immune response (123). Interestingly, CD8 deficient mice show a decrease in macrophage infiltration and adipose tissue (124), suggesting that CD8^+^ T cells infiltration precedes macrophage accumulation in inflammation. So, CST reduces inflammation, macrophage infiltration and might even influence the adaptive immune response by affecting Treg infiltration and decreasing CD8^+^ T cell infiltration, but that would need to be validated in future experiments.

Although it is increasingly clear that CST exerts anti-inflammatory effects on macrophages, the underlying mechanisms are still largely unknown. A key open question is to which receptor CST binds to exert its effect. This could either be a plasma membrane receptor as well as an intracellular target, since CST can penetrate the cell membrane of neutrophils (76, 77). Another question is which signaling pathways are influenced by CST. Based on experiments with inhibitors in mast cells, CST treatment leads to cellular activation by mobilizing intracellular Ca^2+^ and inducing the production of pro-inflammatory cytokines/chemokines (GM-CSF, CCL2, CCL3, and CCL4) via a mechanism possibly involving G-proteins, phospholipase C and the mitogen-activated protein kinase/extracellular signal-regulated kinase (ERK) (119). However, it is unknown whether these pathways are also responsible for the anti-inflammatory signaling in macrophages by CST.

## Clinical implications of CST

Given its roles in metabolic regulation and immune homeostasis, CST has potential clinical applications as a diagnostic marker and even as a therapeutic target. For example, lower levels of CST have been reported in the blood of patients suffering from T2DM, suggesting that it might be a diagnostic marker for this disease (75, 95, 98). However, it might be more useful to study CST levels relative to other cleavage products of CgA, considering that some of these cleavage products counteract the activities of CST. For instance, PST exerts opposing effects on insulin sensitivity and glucose metabolism compared to CST (58), and increased levels of PST can contribute to T2DM (41). The observed lower levels of CST might well be caused by dysregulation of proteolytic processing of CgA (98), since this could result in a higher ratio of PST to CST. Indeed, an altered processing of CgA has been observed in the microenvironment of tumors. Here CgA cleavage products lead to proangiogenic activity, as cleavage of the N- and C-terminal regions of CgA can activate antiangiogenic (vasostatin) and proangiogenic sites (CST), respectively (1). Further supporting the notion that CgA and its cleavage products can be diagnostic markers for various diseases, is that elevated levels of CgA have been detected in the plasma of patients with neuroendocrine tumors (25), hypertension (99, 100) and various inflammatory diseases, such as RA (6, 101, 102), SLE (6), IBD (53, 54, 103–105) as well as T1DM and T2DM (62, 106–109). However, not all assays used in the aforementioned studies allow to discern full-length from proteolytically processed CgA (125) and it would be very interesting to compare this to levels of unprocessed CgA and its cleavage products.

As described above, studies in mouse disease models have indicated that CST can be used as a therapeutic agent for treatment of various diseases, such as colitis, atherosclerosis and diabetes (42, 75, 78, 98, 121, 125). In particular in T2DM, CST is a promising drug candidate, since it basically targets all characteristics of T2DM and modulates both inflammation and metabolism by lowering blood glucose levels, improving insulin sensitivity and secretion as well as by reducing systemic inflammation (3, 126). Especially the ability of CST to shift macrophage polarization toward an anti-inflammatory phenotype makes it a strong therapeutic candidate for a range of inflammatory diseases, such as chronic inflammation (gastritis and colitis), auto-immune diseases (RA and SLE), hypertension, cancers and even inflammation-induced tumor metastasis (9, 25, 127).

## Concluding remarks

As discussed in this review, CST can decrease inflammation by reducing immune infiltration in inflamed tissues and altering macrophages differentiation into an anti-inflammatory phenotype (42, 75, 78, 98, 121, 125). These effects are already observed at concentrations in the nM range, which corresponds to physiological levels of circulating CST (9). By lowering the production of pro-inflammatory cytokines, CST may suppress inflammatory immune responses and/or might promote the dissolvement of inflammation. As a result, CST could prevent chronic states of inflammation and inhibit exaggerated inflammatory responses normally leading to tissue damage. Although CST exerts primarily anti-inflammatory effects, other cleavage products of CgA have opposing pro-inflammatory effects. Disbalances in the levels of circulating CgA-derived peptides might therefore contribute to various diseases (3). Detection and distinguishing of CgA cleavage products with current ELISA-based assays are imperfect, requiring more sensitive mass spectrometry-based assays instead. The mechanism by which CST (and other CgA cleavage products) is removed from the circulation remains unknown; amongst others, its receptor-binding partners need to be identified for instance by immune precipitation followed by proteomics. These data are required to fully understand the effects of CST and other CgA cleavage products.

Due to their effects on immune homeostasis, CST and other CgA-derived peptides are promising targets for diagnosis and therapy of diseases with an inflammatory component, such as diabetes, cancer and RA. A caveat is that almost all current studies on CgA have been conducted in mice and rats. Translating the findings from rodent to man will be essential and will help understanding and designing future diagnostic and therapeutic strategies.

## Author contributions

EM and GD wrote the manuscript. MN assisted in the literature search. SM and GvdB participated in discussion and reviewed/edited the manuscript.

### Conflict of interest statement

The authors declare that the research was conducted in the absence of any commercial or financial relationships that could be construed as a potential conflict of interest.
